# Microfluidic flow-injection aptamer-based chemiluminescence platform for sulfadimethoxine detection

**DOI:** 10.1007/s00604-022-05216-6

**Published:** 2022-02-23

**Authors:** Yanwei Wang, Simone Rink, Antje J. Baeumner, Michael Seidel

**Affiliations:** 1 Institute of Hydrochemistry, Chair of Analytical Chemistry and Water Chemistry, Technical University of Munich, Lichtenbergstraße 4, 85748 Garching, Germany; 2 Institute of Analytical Chemistry, Chemo- and Biosensors, University of Regensburg, Universitätsstraße 31, 93053 Regensburg, Germany

**Keywords:** 3D mixer, Chemiluminescence, *m*-Carboxy luminol, AuNPs, Aptamer, Sulfadimethoxine

## Abstract

Gold nanoparticle–catalyzed chemiluminescence (CL) of luminol is an attractive alternative to strategies relying on enzymes, as their aggregation leads to significantly enhanced CL signals. Consequently, analytes disturbing such aggregation will lead to an easy-to-quantify weakening of the signal. Based on this concept, a homogeneous aptamer-based assay for the detection of sulfadimethoxine (SDM) has been developed as a microfluidic CL flow-injection platform. Here, the efficient mixing of gold nanoparticles, aptamers, and analyte in short channel distances is of utmost importance, and two-dimensional (2D) and three-dimensional (3D) mixer designs made via Xurography were investigated. In the end, since 2D designs could not provide sufficient mixing, a laminated 3D 5-layer microfluidic mixer was developed and optimized with respect to mixing capability and observation by the charge-coupled device (CCD) camera. Furthermore, the performance of standard luminol and its more hydrophilic derivative *m*-carboxy luminol was studied identifying the hydrophilic derivative to provide tenfold more signal enhancement and reliable results. Finally, the novel detection platform was used for the specific detection of SDM via its aptamer and yielded a stunning dynamic range over 5 orders of magnitude (0.01–1000 ng/ml) and a limit of detection of 4 pg/ml. This new detection concept not only outperforms other methods for SDM detection, but can be suggested as a new flow-injection strategy for aptamer-based rapid and cost-efficient analysis in environmental monitoring and food safety.

**Graphical abstract:**

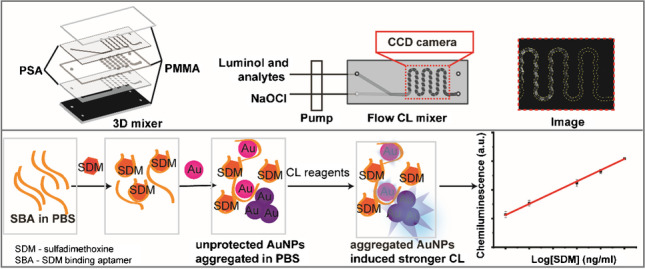

**Supplementary Information:**

The online version contains supplementary material available at 10.1007/s00604-022-05216-6.

## Introduction

Sulfadimethoxine (SDM) is one of the antibacterial drugs which plays a significant role in preventing and treating diseases caused by bacterial infections [1]. However, SDM residues have been found in food and environmental water samples, which not only harm human health through the food chain, but also lead to antibiotic resistance and increase the difficulty of using antibiotics. Therefore, the maximum SDM residue in foodstuffs has been proposed as 100 ng/ml by many countries [2]. Conventional methods for the determination of SDM involve high performance liquid chromatography (HPLC) and gas chromatography-mass spectrometry (GC–MS) [3–5]. A limit of detection lower than 10 pg/ml can be achieved by combining solid-phase extraction with HPLC [6]. However, the instrument is bulky and expensive, and the operation is complex and laborious. The method is very suitable for confirmation with reliability and sensitivity but not for screening large amounts of samples. Other methods (such as colorimetric [7–9], fluorescence [10–12], and electrochemical methods [13–15]) are quick in detection and low cost. Disadvantages for them include the relative low sensitivity, complex labeling process, and narrow linear range. As shown in Table 1, our reported method is simple, rapid, highly sensitive, and cost-effective, as analytical principle was applied a micro-flow injection chemiluminescence (CL) assay which uses gold nanoparticles as CL catalyst and aptamers as selective receptor for SDM.

**Table 1:** Comparison of different methods for SDM detection

Method	Materials	LODs (ng/ml)	Linear ranges (ng/ml)	Advantages	Disadvantages	Reference
HPLC	Sodium dodecylbenzene sulfonate enhanced micro solid-phase extraction	0.59	1–200	High sensitive	Complex operation, expensive instrument	[16]
	Magnetic mixed hemimicelles solid-phase extraction	0.033	-			[6]
Colorimetric	AuNPs, aptamer	50	50–1000	Rapid, visible	Insufficient sensitivity	[7]
	Graphene/nickel@palladium, aptamer	0.7	1–500			[8]
	AuNPs, aptamer	10	10–10^6^			[9]
Fluorescence	Magnetized upconversion nanoparticles,aptamer	0.11	1–9	High sensitive	Complex labeling operations, narrow linear range	[10]
	Cadmium telluride (CdTe) quantum dots	2.24	25–300	Rapid, low cost	Relatively low sensitivity	[11]
	Coordination polymer nanobelt (CPNB), aptamer	10	10–1000			[12]
Electrochemical	Plastic membrane electrode	7.5	-	Rapid	High cost and relatively low sensitivity	[13]
	Molecularly imprinted overoxidized polypyrrole	2.17 × 10^4^	4.65 × 10^4^–1.15 × 10^7^			[14]
	Boron-doped diamond (BDD)	2	10–1.2 × 10^5^			[15]
Micro-flow injection CL	AuNPs, aptamer	0.004	0.01–10^3^	High sensitive, low cost, simple operation		This work

Chemiluminescence (CL) is a luminescence emission mechanism produced by a chemical reaction without the use of an external light source or optical filters. The significant advantages of CL analysis (such as low detection limit, wide linear range, simple instrument, low cost, and fast response speed) make CL a simple, sensitive, and cost-effective analysis technique [17]. Recently, some researchers have applied gold nanoparticles (AuNPs) as catalyst in CL systems [18–20]. Compared with enzymes, they have the advantages of easy preparation and modification, large surface area to volume ratio, and stability [21]. Furthermore, AuNPs have characterized absorbance and visible color [22], and the local plasmon resonance of AuNPs can enhance the local electric field [23] and facilitate the transfer of energy [24]. In our recent work, the synthesis parameters for glucose-reduced AuNPs were optimized as good catalyst for luminol-NaOCl–based CL measurements [25]. Luminol is one of the most applied CL reagents. However, applications are limited by its insolubility under physiological conditions. A water-soluble *m*-carboxy luminol was investigated by the Baeumner group and exhibited a higher CL signal compared to commercial luminol [26].

In modern analytical instruments, manual handling of solutions has been replaced by flow-based injection, which is compatible with computers and can be automatically processed under strict control of reaction conditions. The combination of CL method and flow-injection analysis reduces the analysis time, and obtains high precision and high sensitivity [27]. For CL sensing, continuous flow-injection improves mixing between the luminol and oxidant, which can result in a higher intensity of CL signal than in a cuvette [20]. If luminol, oxidizer, and catalyst are continuously pumped into the microfluidic chip, the flow-based method can emit light continuously. On a microfluidic platform, effective fluid mixing is an essential process, during nanomaterials synthesis, drug delivery, bioreactors, and sample analysis [28]. Mixing can be easily achieved by turbulence of fluid in a macro-system. However, in a microfluidic system, mixing is a time-consuming process. Due to the size of the micro-channel and the low flow rate, the fluid movement is laminar, and the mixing mainly depends on molecular diffusion [29]. Therefore, micromixers are an important part in the microfluidic system. Active mixing which applies an additional effect (such as electric fields, sound waves, and magnetic fields) on the flow field through external equipment can periodically disturb the flow field to improve mixing efficiency [30]. Hence, it takes a short time to complete the mixing. However, complex structures were the bottleneck in the design and manufacture of these mixers. Moreover, additional equipment is required to provide driving force, which greatly increases the cost of the microfluidic chip. The passive mixers involve no external energy, and they change the flow field through geometrical modifications (such as a specially designed flow channel shape), thereby improving the mixing efficiency [31, 32]. Based on their specific structures, a variety of passive mixing methods have been developed, such as spiral micromixers [33], zigzag-shaped channels [34, 35], floor-grooved channels [36], split and recombine (SAR) mixers [37, 38] obstacle-based mixers [39], herringbone mixers [40], T-shaped mixers [41], and convergent-divergent walls [42]. All the above designs can be used in 2D and 3D devices. 3D passive micromixers usually benefit from the spatial structure to produce more effective vortices. However, the fabrication of 3D devices is generally more complicated and more expensive than 2D devices. Xurography is a rapid alternative for conventional microfluidic device fabrication methods. This method attracted attention during the last decade for cheap and rapid prototyping of microfluidic devices without using a cleanroom [43]. The 3D multilayer micromixer using Xurography is an option to fabricate an effective mixer.

The CL signal in microchannels can be imaged by a CCD camera for sensing applications. The possible limitation of these methods is the lack of selectivity. Antibody-based immunoassays have good sensitivity and selectivity; however, the poor stability of antibodies can result in false-negative results [9]. Furthermore, animals are needed for the development or production of polyclonal and monoclonal antibodies. To overcome the above limitations for rapid test systems applicable in the field, aptamers have emerged as new promising recognition biomolecules for analytical applications. Aptamers are single-stranded DNA or RNA oligonucleotides that can specifically bind various target molecules (such as nucleic acids, proteins, metal ions, and other small molecules) with high affinity, selectivity, and sensitivity [44]. Aptamers can be routinely produced in large quantities at low cost by chemical synthesis, avoiding the use of animals for antibody production, and they can be easily modified with different functional groups for labeling or immobilization. Moreover, they are chemically stable, and their shelf life is prolonged. What’s more, aptamer can hybridize with complementary DNA which expands application areas [45, 46]. Given all these attractive features, various aptamer-based analytical CL systems have been developed [47, 48]. However, to our knowledge, there is no homogeneous aptamer assay developed for SDM detection by CL. In the present paper, a microfluidic CL measurement system with AuNPs as catalyst and *m*-carboxy luminol as CL reagent was applied. Synthesized AuNPs aggregated in PBS with an enhanced CL signal and aptamer can inhibit the aggregation. To achieve efficient mixing in limited space, a novel 3D mixer was developed with five layers according to the optimal mixing pattern. The 3D mixer was fabricated by a rapid and low-cost Xurography method. The combination of 3D microfluidic flow-injection with *m*-carboxy luminol and gold nanoparticles as miniaturized chemiluminescence analysis system yielded a stunning dynamic range of 5 orders of magnitude and a very low limit of detection. This method could be applied for continuous monitoring of antibiotics in food or environmental samples.

## Experimental

### Reagents and materials

All reagents were purchased from Sigma-Aldrich (www.sigmaaldrich.com) unless stated otherwise. Poly (methyl methacrylate) (PMMA) sheets (thickness of 0.2 mm) were purchased from modulor material total (Berlin, Germany, www.modulor.de). Double-sided pressure-sensitive adhesive (PSA) tapes (ARcare 90,106) were supplied by Adhesive Research (Glen Rock, PA, USA, www.adhesivesresearch.com). The black PMMA carrier sheets with a thickness of 2 mm were fabricated in our in-house workshop. Erythrosin extra bluish and Fast Green FCF were used to show flow profile. Commercial luminol stock solution (4 × 10^–2^ M) was prepared by dissolving 3-aminophthalhydrazide in 0.10 M sodium hydroxide (NaOH, reagent grade, ≥ 98%, pellets). *m*-Carboxy luminol was provided by the Baeumner group [26], and the stock solution (1 mM) was prepared in carbonate buffer. Carbonate buffer (pH 9.6, 1 L) was prepared by adding 5.76 g of sodium bicarbonate (NaHCO_3_, ACS reagent) and 3.33 g of sodium carbonate (Na_2_CO_3_, anhydrous, ACS reagent) to water. Working solutions of luminol were prepared by diluting the stock solution with carbonate buffer. Working solutions of sodium hypochlorite (NaOCl) were prepared freshly from NaOCl (12% Cl) purchased from Carl Roth (Karlsruhe, Germany, www.carlroth.com). Gold (III) chloride trihydrate (HAuCl_4_ · 3H_2_O) (≥ 99.9%, trace metal basis) and *D*-glucose were used for the synthesis of gold nanoparticles (AuNPs). Sulfadimethoxine (SDM) was prepared in water. The sequence of SDM binding aptamer (SBA) 5’-GAGGGCAACGAGTGTTTATAGA-3’ [12] was synthesized by Eurofins Genomics (Ebersberg, Germany, www.eurofinsgenomics.eu), and SBA was prepared in phosphate buffered saline (PBS, pH 7.4).

### Devices and software

The layout of the mixer was designed with the software CorelDRAW (www.coreldraw.com). The digital cutting plotter (Graphtec CE6000-40) was provided by Graphtec Corporation (Yokohama, Japan, www.graphteccorp.com) to cut sheets into designed layers. Cutting conditions and settings were executed via the Cutting Master 3 from Graphtec Corporation (Yokohama, Japan, www.graphteccorp.com). The pH of solutions was measured with a FiveEasy pH meter FP20 (Mettler Toledo, Columbus, OH, USA, www.mt.com). The CCD camera (16-bit, Model SXV-H9C) was from the Starlight Express Ltd (www.sxccd.com). ImageJ (www.imagej.net) was used to analyze pixel values of pictures taken by the camera. A pump (pump 11, www.harvardapparatus.com) was used to inject the reagents. UV–Vis absorbance spectra for gold nanoparticle suspensions were recorded using a SPECORD 250 PLUS UV/Vis spectrometer (Analytik Jena, Jena, Germany, www.analytik-jena.com). A beam of light with a wavelength ranging from 400 to 900 nm was used for measurement. Disposable polystyrene cuvettes (UV cuvette semi-micro, 1.5–3.0 ml, BRAND GMBH, Germany, www.sigmaaldrich.com) were used for containing samples and reference.

### Chemiluminescence in mixers

**Fig. 1:**
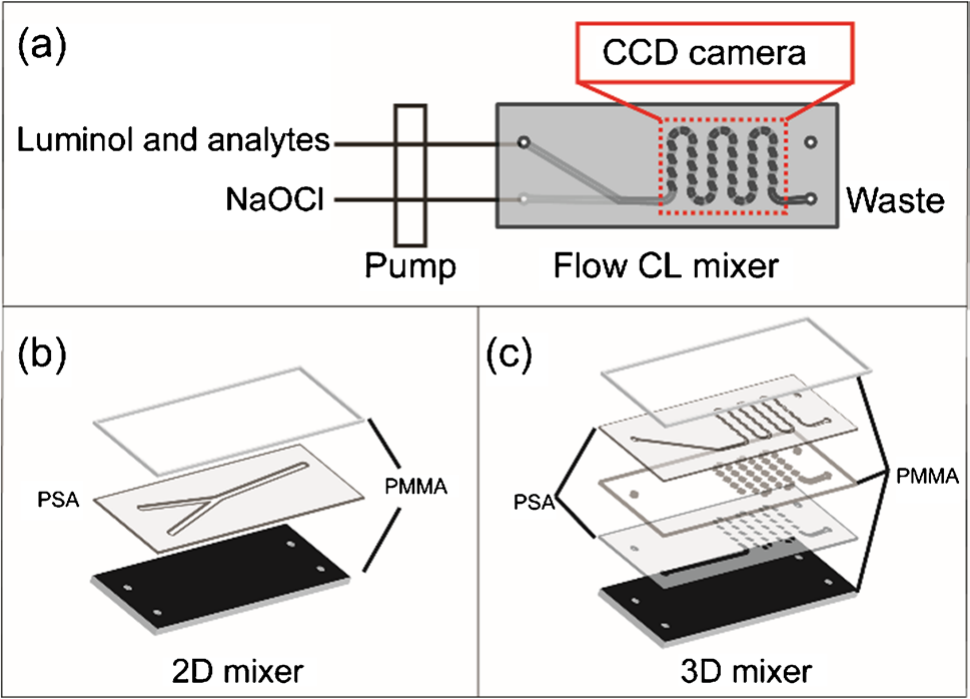
**a** Scheme of chemiluminescence (CL) measurement system: the reagents were pumped into mixer to generate CL signal which was recorded by a CCD camera (the observation area was marked by red dashed lines); **b** scheme of 2D mixer structure with 3 layers; **c** scheme of 3D mixer structure with 5 layers

CL measurement was performed with a CCD camera, equipped with a microfluidic mixer. The chip had two inlets for injecting fluids (luminophore and NaOCl) to the main mixing channel, an observation zone for CCD camera and one outlet, as shown in Fig. 1a. The visible light (425 nm) generated by luminol-NaOCl reaction was recorded by the CCD camera. The CL intensity was the gray value of each pixel, ranging from 0 to 65,536 a.u. (from dark to bright). The microchip was composed of transparent PMMA layers, PSA layers, and one black PMMA carrier with holes for inlets and outlet. The 2D micromixer was composed of three layers (Fig. 1b). The transparent PMMA cover allows generated light to pass through. The middle PSA layer with different channels offers a place for reagents mixing and CL generation. The black PMMA carrier can shield the light from the environment and offers a dark blank. For 3D mixer (Fig. 1c), additional PMMA and PSA layers were added to provide more mixing space and cause turbulence. The *m*-carboxy luminol and commercial luminol from Sigma-Aldrich were compared by flow injection method in the 3D mixer. The concentration of luminophore was 0.5 mM, and the pH was adjusted by carbonate buffer to 9.6. Luminophore was mixed with 1% NaOCl in 3D mixer by a pump with a flow rate of 10 ml/h. The signal was recorded by a CCD camera with an exposure time of 30 s and repeated 25 times.

### Homogeneous detection of sulfadimethoxine

**Fig. 2:**
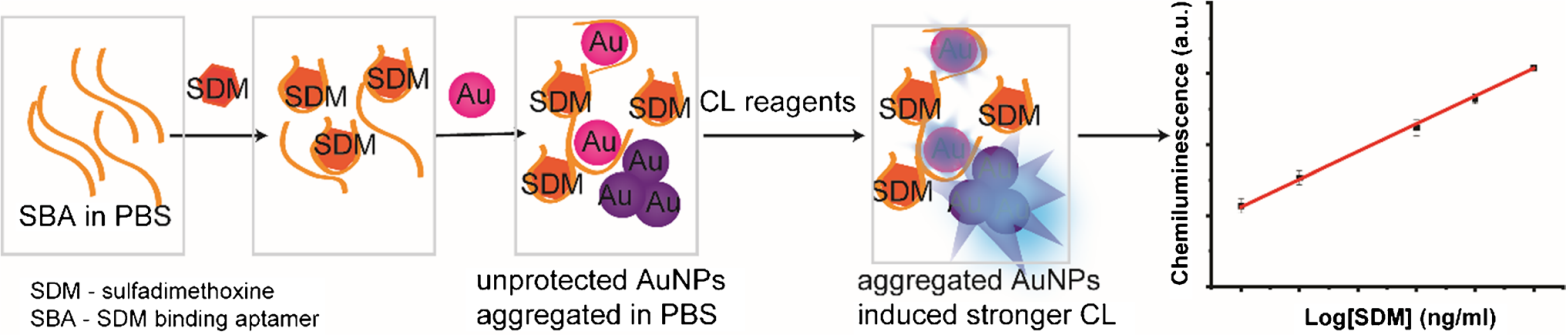
Schematic illustration of homogeneous chemiluminescence detection of SDM using AuNPs probe

AuNPs were synthesized in an automatic way through a single-phase reaction using glucose as reducing agent. The synthesized AuNPs were used as catalyst in luminol-NaOCl system, and the synthesis parameter had been optimized by our group [25]. Briefly, 0.05% HAuCl_4_, 2 mM NaOH and 1 M glucose were mixed in a 3D microreactor in a flow rate of 0.5 μl/s, 2.5 μl/s, and 2.5 μl/s, respectively. The synthesized AuNPs were quasi-spherical with a diameter of 15.32 ± 1.09 nm and aggregated AuNPs caused by salt can enhance the CL signal [25]. The principle for homogeneous detection of SDM was shown in Fig. 2. The SBA can protect AuNPs from aggregation in PBS. When there is SDM in the system, it will bind with SBA with a strong affinity. In this case, there will not be enough SBA to stabilize the AuNPs causing aggregation and thus a stronger CL signal. If SDM is absent, SBA will adsorb on the surface of AuNPs and inhibit the aggregation. Therefore, the dispersed AuNPs generate a weak CL signal. All reagents were continuously pumped into a 3D mixer where CL signal was generated and recorded. One hundred microliters of 1 μM aptamer (in PBS) was mixed with 100 μl SDM at different concentrations. Then, 200 μl synthesized AuNPs were added to the mixture and incubated for 5 min. Subsequently, 200 μl of 0.1 mM m-carboxy luminol was added to the solution and incubated for 30 min. The mixture was mixed with 0.2% NaOCl in 3D mixer by a pump with a flow rate of 20 ml/h. The signal was recorded by a CCD camera with an exposure time of 10 s and repeated 3 times. The protocol for measuring a sample is described in Supplementary material.

## Results and discussion

### Design of mixers

**Fig. 3:**
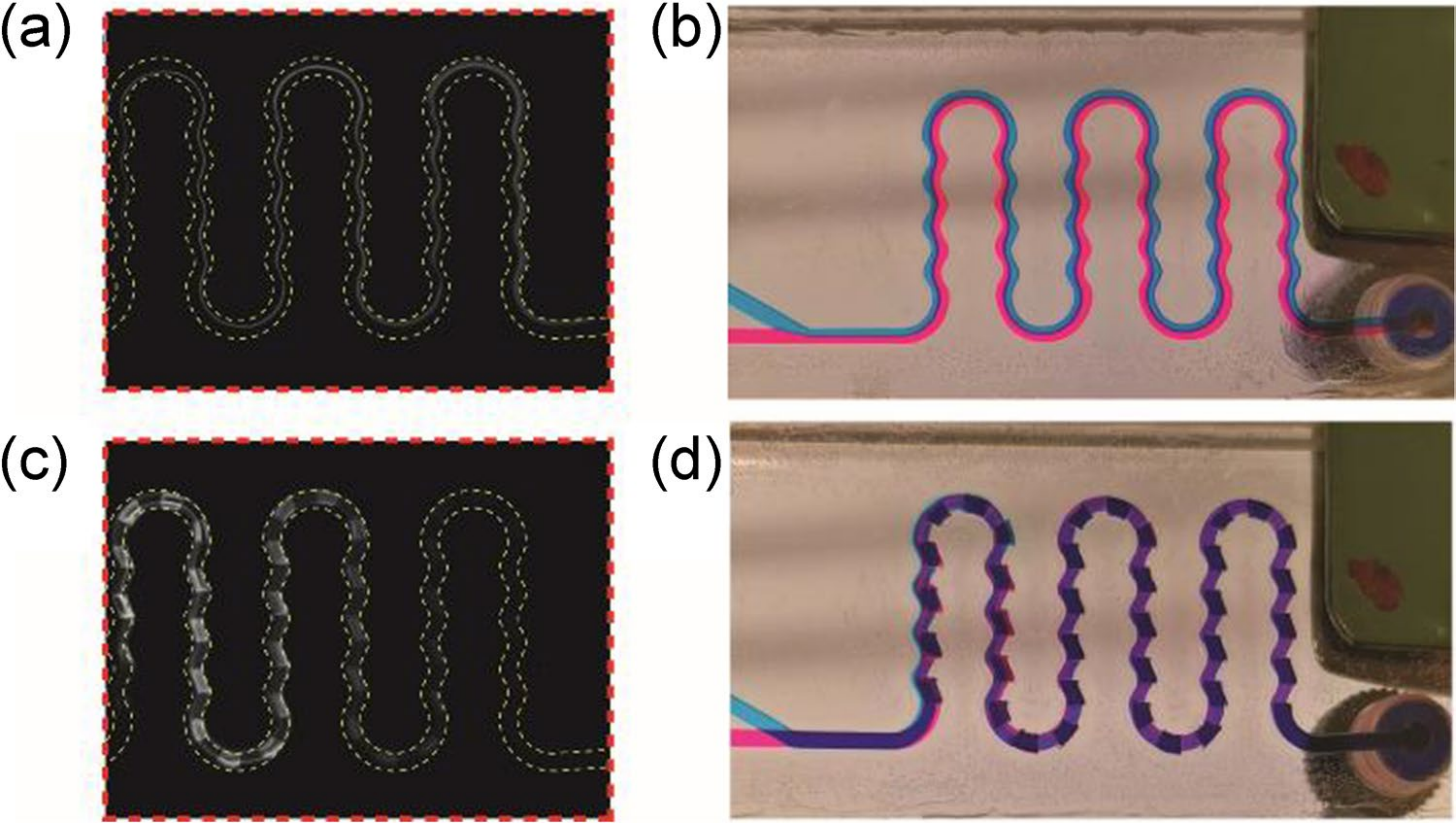
Images of chemiluminescence reaction in **a** 2D and **c** 3D mixer. **b** and **d** The mixing profile of 2D and 3D mixer which have the same structure as (**a**) and (**c**), respectively

For better mixing, the structure of mixer was optimized both in 2D and 3D which are detailedly described in Supplementary material. The design was then transferred to the CL chip. The CL signal obtained with the wavy line pattern (WL) in the 2D and 3D chip were compared. For the 2D chip, the signal only emerged in the center of the mixing channel. The signal was low at the beginning and increased along the channel. The signal was still high at the end of the channel (Fig. 3a). This can be explained by inefficient mixing taking place only at the interface of both liquid streams. With progression of the liquid flow along the channel, mixing becomes more effective and a higher CL signal was obtained as shown in Fig. 3b. In contrast, with the 3D chip, the generated light filled the complete mixing channel and higher signals were obtained at the beginning (Fig. 3c). Afterwards, the signal decreased and then almost disappeared at the end indicating the mixing was well, and all the reagents were consumed. As shown in Fig. 3d, the mixing occurs across the whole channel, and it was efficient.

### Comparison of luminol and m-carboxy luminol in 3D mixer

The commercial luminol and *m*-carboxy luminol were injected in a 3D mixer to react with NaOCl, and the result was shown in Fig. S7. With commercial luminol, the mean signal was 3787 a.u. with standard deviation of 852 a.u. When commercial luminol was substituted by *m*-carboxy luminol, the CL signal was increased more than 10 times to 55,914 a.u. Moreover, the signal was more stable with a standard deviation of 303 a.u.

### Homogeneous detection of sulfadimethoxine

**Fig. 4:**
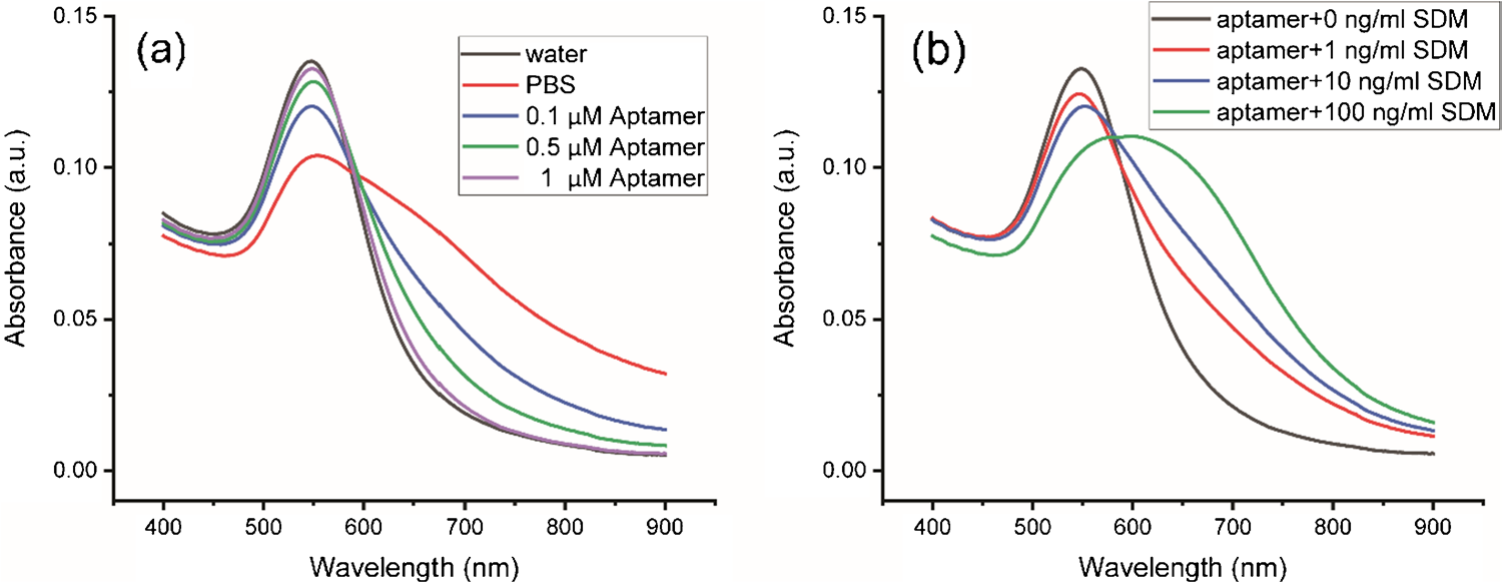
**a** UV − Vis spectra of AuNPs in water, PBS, and various concentrations of aptamer in PBS; **b** UV − Vis spectra of AuNPs solution in 1 μM aptamer (in PBS) and various concentrations of sulfadimethoxine (in water)

The synthesized AuNPs were stable in water and have a strong plasmon absorption at 548 nm. After adding PBS, the absorption spectrum of AuNPs was broad and featureless, indicating that AuNPs aggregated as shown in Fig. 4a. After the aptamer was introduced, the absorption spectrum was restored close to that of AuNPs in water which indicated that aptamers can protect AuNPs from aggregation [49]. The amount of aptamer was investigated. Aptamers at low concentration do not adequately stabilize AuNPs in PBS. However, if the aptamer concentration is too high, SDM could bind with extra aptamer, and the detection is not sensitive. Experimental results showed that the optimal concentration of aptamer was 1 μM. UV–Vis absorption spectrum analysis was performed to check whether the SDM was bound to the aptamer in the system. As shown in Fig. 4b, without SDM, the absorption of AuNPs was high and narrow, indicating the dispersion of AuNPs. As the concentration of SDM increased, the intensity of plasmon absorption decreased. Moreover, the absorption spectra were broader and shifted to a high value, indicating aggregation of AuNPs. This phenomenon can be explained by the binding of SDM and aptamer, and the remaining SBA was not enough to stabilize AuNPs in PBS.

**Fig. 5:**
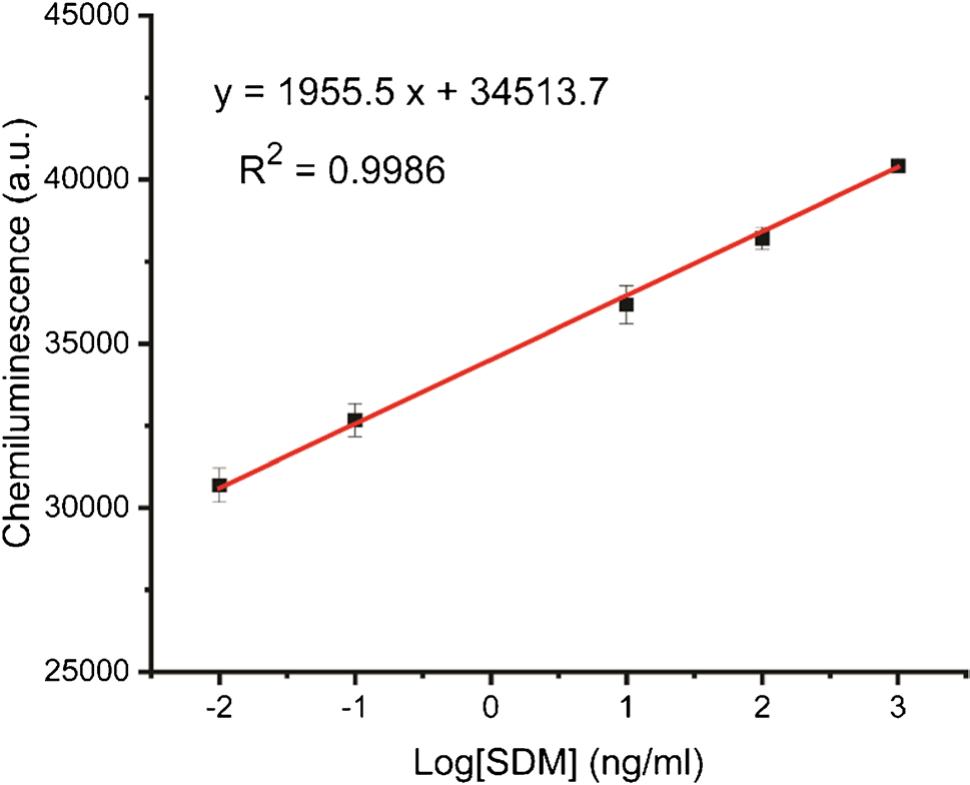
The calibration plots of chemiluminescence intensity versus the logarithm of sulfadimethoxine concentration using AuNPs catalyzed chemiluminescence with *m*-carboxy luminol in a homogeneous aptamer-based assay in a flow-based 3D mixer chip, *n* = 3

Under the optimal detection conditions, the CL signal in the presence of various concentrations of SDM were detected and shown in Fig. S8. As shown in Fig. 5, the CL signal increased with the increasing concentration of SDM. When SDM concentration changed from 0.01 to 1000 ng/ml, there was a linear relationship between the CL signal and the logarithm of the SDM concentration. The linear regress equation can be expressed as *y* = 1955.5 *x* + 34,513.7 (*y* is the intensity of CL signal, *x* is the logarithm of SDM concentration) with a correlation coefficient of 0.9986 (*n* = 3, *m* = 5). The detection limit that is taken to be three times the standard derivation in the blank solution (29,302.6 ± 179.5) was found to be 0.004 ng/ml.

Compared with other reported work, it was shown that micro-flow injection CL method is a very sensitive method for SDM by using aptamers, gold nanoparticles, and *m*-carboxyl luminol. The *m*-carboxyl luminol with higher CL quantum yield further enhanced the CL signal difference [26]. Moreover, the water-soluble *m*-carboxyl luminol can be more uniformly dissolved in the solution; therefore, the signal was more stable with a lower standard deviation, which leads to the lower limit of detection. The wide linear range of the calibration curve could be attributed to the huge catalytic difference between dispersed AuNPs and aggregated AuNPs.

### Recovery determination using water sample

To evaluate the applicability of the developed method, 50 ng/ml SDM was spiked in water. There was no pretreatment for the sample. One hundred–microliter sample was directly mixed with 100 μl of 1 μM aptamer (in PBS). Then 200 μl synthesized AuNPs were added and incubated for 5 min. After that, 200 μl 0.1 mM m-carboxy luminol was added to the solution and incubated for 30 min. Finally, one-third above-mentioned mixture (200 μl) was mixed with 200 μl 0.2% NaOCl in the 3D mixer by a pump with a flow rate of 20 ml/h. 3 continuous images were recorded by a CCD camera with an exposure time of 10 s for each image. The recovery (%) for the water sample was shown in Table 2. The recovery was 96.8% with relative standard deviation (RSD) of less than 1%. These results indicate that our method has excellent accuracy and stability for the detection of SDM in water.

**Table 2:** Recovery results of water sample (*n* = 3)

Spiked (ng/ml)	Detected (ng/ml)	Recovery (%)	RSD (%)
50	48.42 ± 0.16	96.8 ± 0.31	0.32

## Conclusions

In this paper, a 3D microfluidic flow-injection platform with AuNPs-catalyzed and *m*-carboxy luminol enhanced chemiluminescence for aptamer-based homogeneous assays was developed. This new detection concept is superior to other methods for SDM detection with high sensitivity. It offers a rapid and cost-efficient analysis method in environmental monitoring where antibiotics often occur at concentrations from ng/L to μg/L [50]. Not only for antibiotics but this approach can also be suggested as a new flow-injection strategy for the detection of analytes whose aptamers have been confirmed. For application in physiological conditions, detection of analyses in complex matrices with purification systems still needs to be explored [51].

## Supplementary Information

Below is the link to the electronic supplementary material.

Supplementary file1 (DOCX 1212 KB)
