# Supplementary material for: Microfluidic flow-injection aptamer-based chemiluminescence platform for sulfadimethoxine detection
Source: Mikrochim Acta. 2022 Feb 23;189(3):117. doi: 10.1007/s00604-022-05216-6 (PMC8866360; doi:10.1007/s00604-022-05216-6)
Supplement: Supplementary file 1 — Supplementary file1 (DOCX 1212 KB) [file 604_2022_5216_MOESM1_ESM.docx]

Electronic Supplementary Material

Microfluidic flow-injection aptamer-based chemiluminescence platform for sulfadimethoxine detection

**Yanwei Wang ^1^, Simone Rink^2^, Antje J. Baeumner^2^ and Michael Seidel^1^***

1. Institute of Hydrochemistry, Chair of Analytical Chemistry and Water Chemistry, Technical University of Munich, Elisabeth-Winterhalter-Weg 6, 81377 Munich, Germany.
2. Institute of Analytical Chemistry, Chemo- and Biosensors, University of Regensburg. Universitätsstraße 31, 93053 Regensburg (Germany)

***** Correspondence: E-mail: Michael.Seidel@mytum.de, Tel: +49 89 2180 78252, Fax: +49 89 2180 78255

**Protocol**

**Material**

*Consumables:*

- Gold (III) chloride trihydrate (HAuCl_4_ · 3H_2_O) (≥ 99.9%, trace metal basis)
- *D*-glucose
- NaOH
- Carbonate buffer (pH 9.6, 5.76 g/L NaHCO_3_, and 3.33 g/L Na_2_CO_3_ )
- Phosphate buffered saline (PBS, pH 7.4).
- *m*-carboxy luminol (1 mM) prepared in carbonate buffer
- NaOCl prepared freshly from NaOCl (12% Cl)
- SDM binding aptamer (SBA) 5’-GAGGGCAACGAGTGTTTATAGA-3’ prepared in PBS

*Lab equipment:*

- CCD camera
- Computer
- Pump
- Syringe

*Software:*

- ImageJ
- SXV_Hmf_usb ([www.sxccd.com](http://www.sxccd.com))

**Synthesis of AuNPs**

0.05% HAuCl_4_, 2 mM NaOH and 1 M glucose are mixed in a 3D microreactor in a flow rate of 0.5 µl/s, 2.5 µl/s, and 2.5 µl/s, respectively. The synthesized AuNPs are collected.

**Measurement of samples**

1. 100 µl 1 µM aptamer (in PBS) mix with 100 µl sample.
2. 200 µl synthesized AuNPs are added and incubate for 5 min.
3. 200 µl 0.1 mM *m*-carboxy luminol is added to the solution and incubates for 30 min.
4. One-third above-mentioned mixture (200 µl ) is mixed with 200 µl 0.2% NaOCl in a 3D mixer by a pump with a flow rate of 20 ml/h.
5. 3 continuous images are recorded by a CCD camera with an exposure time of 10 s for each image.
6. The images are analyzed by ImageJ to get the average pixel value (between 0 to from 0 to 65,536 a.u.)

Design of 2D mixers

For realization of better mixing, seven geometrical structures were applied and comparative analysis for these different micromixers have been done. The reagents can only mix in the middle of the channel for line pattern (LP) mixer and the mixing was not efficient ( **Fig. S 1a**). Tesla pattern (TP) mixer was recommended considering high mixing performance and easy fabrication due to the planar structure [1]. However, the mixing liquid did not fill the complete channel structure as half of the channel remained dark. (**Fig. S 1b**). Similar was observed when the split and recombine pattern (SAR) mixer was applied (**Fig. S 1c**). This is probably due to the two channel which merge and separate again, which generates an inhomogeneous pressure distribution guiding the mixing liquid along the path of least resistance. Therefore, mixers with only one channel were designed. For the convergent-divergent pattern (CD) mixer, the channel was symmetrical, only half of the channel showed bright light generation (**Fig. S 1d**). Other designs such as the zigzag pattern (ZP) and the obstacle-based pattern (OB) favored small bubble entrapment (**Fig. S 1e** and **f**). The mixer with wavy line pattern (WL) showed a better result as most of the channel lighted up and no bubbles were trapped (**Fig. S 1g**). To confirm the efficiency of 2D mixing, the same design (WL) was applied to a transparent chip with dyes to illustrate the flow profile. The chip had two inlet channels for directing the dyes (green and pink) to the main WL mixing channel. As shown in **Fig. S 2**, the dye flow of green and pink went along the channel separately and only the interface of the two dyes changed the color to purple. Although the mixing channel was prolonged 2-times, no complete mixing was achieved.


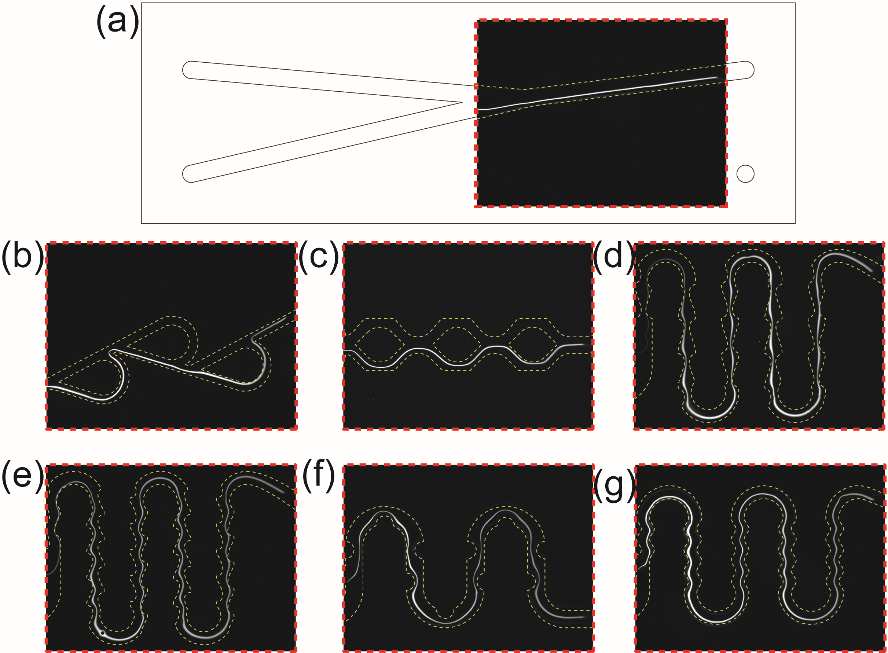


**Fig. S 1** Different designs of 2D micromixer (yellow dash line) and images recorded by a CCD camera (black area with bright light): (a) design of central PSA layer with two inlets and one outlet with line pattern (LP), the area with red dash line can be recorded by a CCD camera; (b) Tesla pattern (TP); (c) Split and recombine pattern (SAR); (d) Convergent-divergent pattern (CD); (e) Zigzag pattern (ZP);(f) Obstacle-based pattern (OB) and (g) Wavy line pattern (WL)


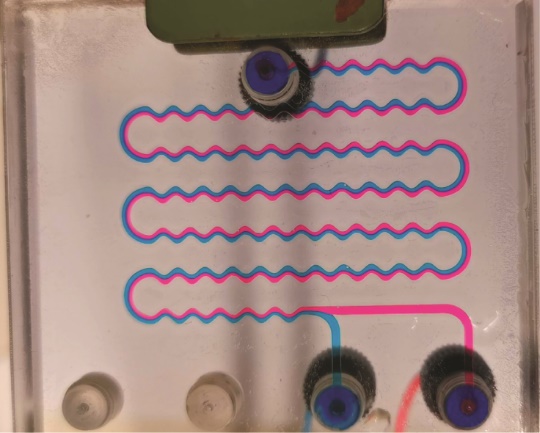


**Fig. S 2** Image of 2D mixing chip with dyes showing flow profile

Design of 3D mixers

As 2D mixers cannot manage the efficient mixing according to the limited space, a 3D mixer was designed with WL for better mixing performance. The structure is shown in **Fig. S 3**. This 3D mixer was composed of two pressure-sensitive adhesive tape (PSA) layers and three PMMA layers. The two PSA layers with an inlet channel for injection and a WL mixing channel were designed for mixing. A PMMA layer with holes was laminated between two PSA layers to expand the mixing space. Various designs of these three layers were compared afterwards. Then, these channels were closed by a transparent PMMA cover and a black PMMA carrier.


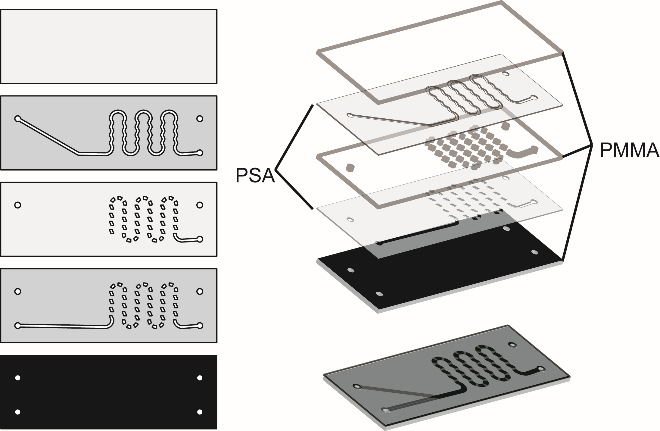


**Fig. S 3** Structure of 3D micromixer for chemiluminescence

The thickness of the central PMMA layer was optimized in transparent 3D mixers using dyes to show the flow profile. For better comparison, the mixing channel was 2-times longer of the length of the CL chip channel. Different thickness of 0.08 mm, 0.17 mm and 0.25 mm were compared. The thicker layer with holes offers more space for mixing, hence the mixing performance was better as shown in **Fig. S 4**. Due to the limitation of the cutting plotter, much thicker PMMA layer cannot be applied, and the thickness was chosen as 0.25 mm.


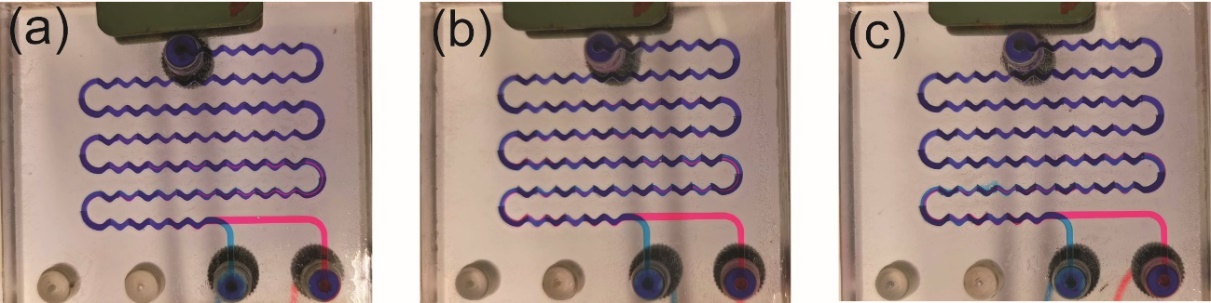


**Fig. S 4** Images of 3D mixer with different thickness of central PMMA layer: (**a**) 0.08 mm; (**b**) 0.17 mm; (**c**) 0.25 mm

Three different structures were compared in transparent 3D mixers, and the sectional view schemes and top view images were shown in **Fig. S 5**. For the first design, the layers with injection channels for two kinds of dyes were WL pattern without obstacle. In this case, liquid can be mixed in the central PMMA layer. However, both flows prefer to run parallel along the channel and show no motivation to mix causing inefficient mixing at the end of channel (**Fig. S 5a**). For the second design, both injection channels were blocked alternately. The two dyes were forced to flow up and down along the channel barriers improving the overall mixing. As shown in **Fig. S 5b**, mixing was achieved, and the color changed to purple already in the middle of the channel. Unfavorable for this design was small bubble entrapment in the upper channel blocked behind the obstacle which were not easy to be flushed out. Moreover, the slight opacity of the PSA layer would hinder the recording of CL light. Therefore, a third design without obstacles in the upper layer channel, blocking only the lower injection layer, was applied. As the image shows, the mixing was finished earlier with more obstacles (**Fig. S 5c**). Different flow rates were applied in the optimal designed 3D mixer. As shown in **Fig. S 6**, with a lower flow rate of 5 ml/h, the mixing was efficient in the mixer. Increasing the flow rate, increases the mixing abilities and for a flow rate of 40 ml/h the mixing was finished even with one fifth of the mixing channel length.


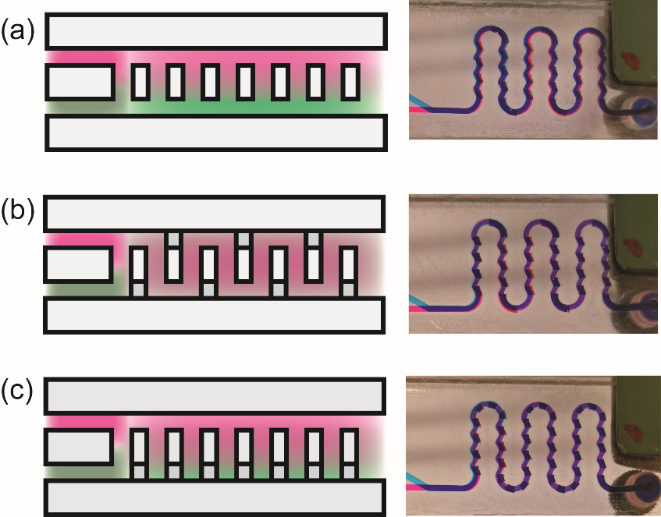


**Fig. S 5** Different designs of 3D structure of micromixer and images recorded with dyes showing flow profile (sectional view schemes on the left and top view images on the right).


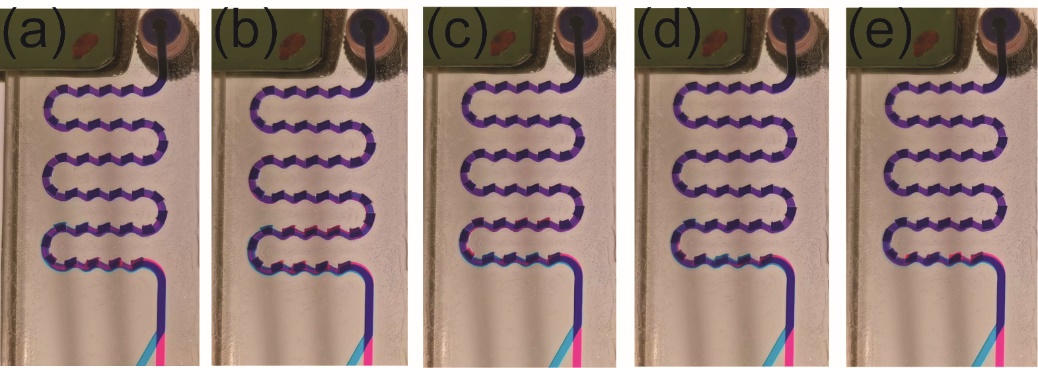


**Fig. S 6** Mixing with different flow rate: (**a**) 5 ml/h; (**b**) 10 ml/h; (**c**) 20 ml/h; (**d**) 30 ml/h; (**e**) 40 ml/h


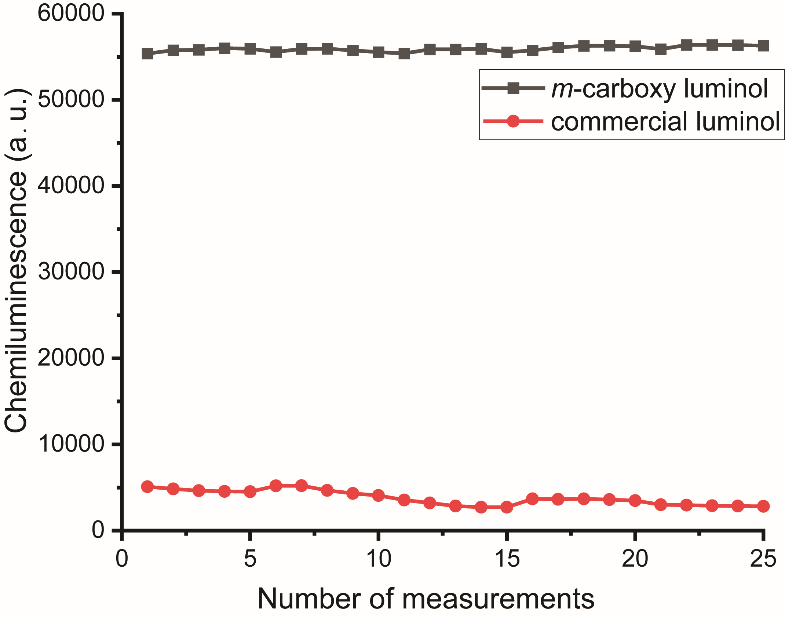


**Fig. S 7** Comparison of chemiluminescence signal in 3D mixer for commercial luminol and m-carboxy luminol. The concentration of both luminophores was 0.5 mM (pH 9.6) mixed with 1% NaOCl. The measurement was repeated 25-times, respectively

Pictures were recorded by CCD camera when different concentrations of SDM were mixed with 1 µM aptamer in PBS, synthesized AuNPs, and CL reagents. The results were shown in **Fig. S 8**. Since the dispersed AuNPs have excellent catalytic activity under the optimal synthesis parameters, the mixture without SDM also generates strong light signals of about 30,000 a.u.. Therefore, the naked eye cannot distinguish the difference in signal strength because all signals were very high. Signals were subtracted by 30,000 a.u. to make the difference easy to distinguish. As the signal was an average value, there were still some white pixels whose value exceed 30,000 a.u. left. The left signals were doubled to enhance the difference. In this case, it can be seen that the CL signal increases with the increase of the SDM concentration. For further analysis with software, values of original pictures were used.


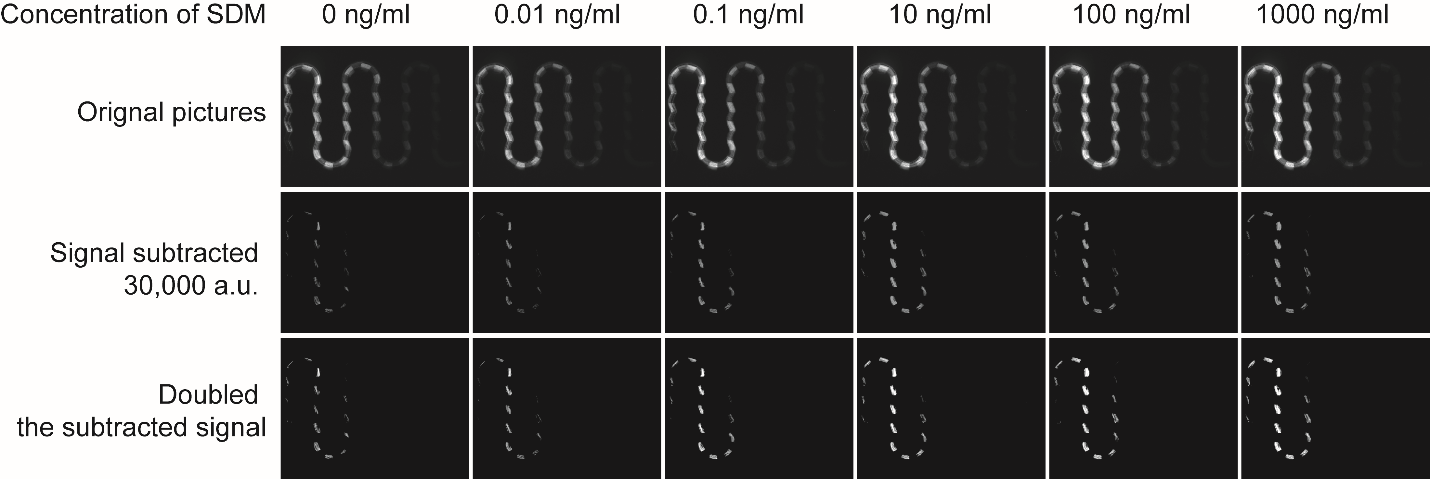


**Fig. S 8** Pictures recorded by the camera with different concentrations of SDM (0 ng/ml to 1000 ng/ml).

Reference

1. Raza, W., Hossain, S., & Kim, K. Y. (2020). A Review of Passive Micromixers with a Comparative Analysis. *Micromachines (Basel), 11*(5), <https://doi.org/10.3390/mi11050455>.
